# Adapting and pilot testing a tool to assess the accessibility of primary health facilities for people with disabilities in Luuka District, Uganda

**DOI:** 10.1186/s12939-024-02314-0

**Published:** 2024-11-13

**Authors:** Islay Mactaggart, Andrew Sentoogo Ssemata, Abdmagidu Menya, Tracey Smythe, Sara Rotenberg, Sarah Marks, Femke Bannink Mbazzi, Hannah Kuper

**Affiliations:** 1 The International Centre for Evidence in Disability, London School of Hygiene & Tropical Medicine, Keppel Street, London, WC1E 7HT UK; 2 Medical Research Council, Uganda Virus Research Institute and London School of Hygiene & Tropical Medicine Uganda Research Unit, Entebbe, Uganda; 3 Division of Physiotherapy, Department of Health and Rehabilitation Sciences, Stellenbosch University, Cape Town, South Africa; 4 Department of Global Health and Development, London School of Hygiene and Tropical Medicine, London, United Kingdom

**Keywords:** Primary health, Accessibility, Disability, Inclusive health, Uganda

## Abstract

**Background:**

People with disabilities frequently experience barriers in seeking healthcare that lead to poorer health outcomes compared to people without disabilities. To overcome this, it is important to assess the accessibility of primary health facilities – broadly defined to include a disability-inclusive service provision – so as to document present status and identify areas for improvement. We aimed to identify, adapt and pilot test an appropriate tool to assess the accessibility of primary health facilities in Luuka District, Uganda.

**Methods:**

We conducted a rapid literature review to identify appropriate tools, selecting the Disability Awareness Checklist (DAC) on account of its relative brevity and development as a sensitization and action tool. We undertook three rounds of adaptation, working together with youth researchers (aged 18–35) with disabilities who then underwent 2 days of training as DAC facilitators. The adapted tool comprised 71 indicators across four domains and 12 sub-domains. We also developed a structured feedback form for facilitators to complete with healthcare workers. We calculated median accessibility scores overall, per domain and per sub-domain, and categorised feedback form suggestions by type and presumed investment level. We pilot-tested the adapted tool in 5 primary health facilities in one sub-district of Luuka, nested within a pilot healthcare worker training on disability.

**Results:**

The median overall facility accessibility score was 17.8% (range 12.3–28.8). Facility scores were highest in the universal design and accessibility domain (25.8%, 22.6–41.9), followed by reasonable accommodation (20.0%, 6.7–33.3). Median scores for capacity of facility staff (6.67%, 6.7–20.0), and linkages to other services were lower (0.0%, 0–25.0). Within the feedback forms, there were a median of 21 suggestions (range 14–26) per facility. Most commonly, these were categorised as minor structural changes (20% of suggestions), with a third categorised as no (2%) or low (33%) cost, and the majority (40%) medium cost.

**Conclusions:**

Overall accessibility scores were low, with many opportunities for low-cost improvement at the facility level. We did not identify any issues with the implementation of the tool, suggesting few further adaptations are required for its future use in this setting.

**Supplementary Information:**

The online version contains supplementary material available at 10.1186/s12939-024-02314-0.

## Background

Access to quality primary health care is essential for the health and wellbeing of all people, including all people with disabilities. However, people with disabilities in both high- and low- income settings frequently experience barriers that lead to inequities in primary healthcare contact, utilisation and outcomes compared with others [1–5]. These barriers include a wide range of physical, attitudinal, logistical, financial and informational factors that hamper access to care [3, 6]. Together, they contribute to lower immunization, treatment and preventative screening rates [7–9], and an estimated 2 fold mortality rate among people with disabilities compared to people without [10]. There are many intersecting factors required to overcome these inequities, integral to which is the development of well-financed disability-inclusive primary health systems where people with disabilities are expected, accepted and connected [5]. A fundamental component of this is improving accessibility of primary health facilities themselves. This includes physical accessibility (e.g. ramps or non-slip flooring), but also the accessibility of information systems (e.g. signage, health educational material), availability of referrals and follow up care, and a disability-inclusive primary health-care workforce informed about and responsive to the needs of patients with disabilities [5, 6].

The majority of people with disabilities live in low or middle income countries (LMICs), where constraints in governance and financing that affect health system’s service delivery and affordability are common, and investment in disability-inclusive healthcare is generally low [6, 11]. Better evidence on the accessibility of primary health facilities is required, to identify practical and cost-effective solutions to the common barriers people with disabilities experience that negatively affect their health. Engaging healthcare workers and people with disabilities directly in such assessments may catalyse this progress, as collaboration may highlight to the healthcare workforce where they can improve their services in line with the priorities of their patients. Accessibility audits are common practice in transportation, websites, and other public spaces to assess the state of disability inclusion and comply with local laws around access for people with disabilities [12–14]. However, the accessibility of the health sector is relatively understudied, with no internationally agreed accessibility standards or best practice tools.

In this pilot study, we sought to identify, adapt and pilot test an appropriate tool to assess the accessibility of primary health facilities in one sub-district of Luuka District in the Busoga sub-region of Eastern Uganda, within a pilot healthcare worker training on disability inclusion.

## Methods

### Study setting

Luuka is a rural district in Eastern Uganda, with an estimated disability prevalence (ages 5+) of 11.7% [15]. There are 43 active primary health facilities in the district, serving an estimated population of 238,000 people. The primary health system includes village level health teams, and three levels of health centres: HC II, HC III and HC IV (Supplementary File Table 1). Health care is provided by a mix of public and private not-for-profit providers, all registered by the district health office [16].

### Identifying an appropriate tool

To identify potential tools, we conducted a rapid review of the existing published literature, supplemented by consultation with experts to identify relevant tools from the grey literature. We searched PubMed in January 2023 using the search string ((“primary health”) AND (accessibility) AND (disability) AND (audit OR checklist OR questionnaire)), with no exclusions by language or date. Only four tools were identified in the peer-reviewed literature that had been developed or tested in LMICs [17–20]. Two further tools were identified in the grey literature as recently developed tools designed for use in LMICs and based on international accessibility standards [21, 22]. We extracted data on each tool’s origin, framework or development background, included domains and number of questions, response options, scoring methodology and previous pilot testing.

### In selecting the tool for use, we considered the following criteria

Relevance: applicable to assessing awareness and practices related to disability inclusivity.

Comprehensiveness: covers a broad range of aspects pertinent to disability accessibility aligning with our research goals.

The Disability Awareness Checklist (DAC) was selected for adaptation, given its relative brevity compared to other tools, its background as a disability sensitisation and action tool, and development in consultation with health-service users with disabilities and in alignment with international frameworks [22]. The selection of the DAC tool was made collaboratively by the research team, which includes experts in disability studies and public health. The choice was informed by the DAC’s applicability to our study context in relation to its relevance and comprehensiveness to identify basic gaps in accessibility and service delivery.

The DAC was developed in South Africa to be used by primary healthcare workers or community representatives, allowing healthcare workers to assess their own facilities and identify areas for simple improvements [22–24]. It includes four domains of disability inclusion: universal design and accessibility; reasonable accommodation; capacity of facility staff to identify and support people with disabilities; and linkages to disability and rehabilitation services. These are split into 5 sub-domains and 59 indicators. Key accessibility measurements (e.g. useable width of doors or corridors, and slope of any ramps) are captured. The DAC has not previously been used in a low-income setting and we note that there are currently no published statistics or comprehensive studies specifically documenting the use of the DAC due to the relatively recent development and implementation of the tool. Despite the lack of published data, the DAC was selected for our study based on its relevance and applicability to our research objectives. The use in our study aims to contribute to the growing body of knowledge and evidence around its effectiveness.

### Adapting the DAC

We undertook three rounds of adaptation of the DAC to maximise its context relevance, prior to pilot testing. In Round 1, we examined the tool’s face validity in the Ugandan context in consultation with eight youth researchers (ages 18–35) participating in the MRC/UVRI & LSHTM Uganda Research Unit capacity building project Disabled Youth Researchers. The youth researchers, themselves all Ugandan healthcare users with disabilities, reviewed the individual questions and suggested adaptations based on the Ugandan primary health sector. For example, questions on guide dogs (not commonly available in Uganda) were removed, and questions on public transport adapted. In Round 2, we created an “Accessibility Standards Minimums” reference document (Supplementary File Appendix 1). In this, we extracted relevant accessibility standards from two local guidelines, the Uganda National Association of Persons with Disabilities Accessibility Standards 2010, and the Ugandan Government 2019 National Building Accessibility Standards for Persons with Disabilities Code [25, 26] and compared these to the adapted DAC questions, updating measurement criteria for the latter accordingly. In Round 3, we completed a detailed round-table review of the draft adapted tool with members of the study team (IZM, AS, FMB, AM) and the youth researchers.

The adapted tool includes 71 indicators across four domains and 12 sub-domains (summarised in Table 1, full tool in Supplementary File Appendix 2).

**Table 1:** List of domains, sub-domains and number of indicators within each section of the adapted tool

Domain	Sub-Domain	No. Indicators within Sub-Domain
Universal Design and Accessibility	Entrance to services	7
	Reception, corridors and waiting rooms	19
	Toilets	5
	**Total**	**31**
Reasonable accommodation of people with disabilities	Information and communication	7
	Assistance and support	8
	**Total**	**15**
Capacity of facility staff to identify and support people with disabilities	Awareness and accessibility training (all)	4
	Emergency training for at least one staff member on duty	2
	Healthcare worker training (at least some staff)	3
	Other training and support capacity	4
	**Total**	**13**
Linkage to disability and rehabilitation services	Disability process data	4
	Annually updated referral processes	5
	Direct service linkages	3
	**Total**	12
**Total**		**71**

Additionally, we developed a structured feedback form (Supplementary File Appendix 3) for facilitators to complete with healthcare workers during a debriefing session, documenting identified gaps and proposed areas for improvement in accessibility of the facility.

### Pilot testing the adapted tool

The eight youth researchers from the Disability Youth Investigates research programme (DYI), a programme to build capacity amongst researchers with disabilities, underwent a 2-day DAC facilitator training led by IZM, ASS and AM at the MRC/UVRI & LSHTM Uganda Research Unit facility in Entebbe in August 2023. Topics included preparing for and completing the DAC, processes and logistics, and practice at two nearby health facilities.

The pilot version of the data collection form was built on REDCap [27] and administered using encrypted and password-protected tablets. To inform feasibility, additional fields were built into the form to document data availability, issues with data capture or interpretation, and time stamping. All infrastructure measurements were taken in a straight line (180°) at the narrowest point as this is the useable space for the patient. For long ramps (over 2 m), 2–3 measurements were taken at various points along the length, and the steepest angle was recorded. GPS locations were recorded for the locations of (a) the facility (b) the nearest public transport drop off (bodaboda stage) (c) the nearest private taxi drop off.

The pilot was nested within a pilot healthcare worker training programme [28] as part of a broader research project aimed at improving access to inclusive health for persons with disabilities in Luuka District [29].

Six of the eight youth researchers were selected to participate in the pilot as trainee facilitators, which included 5 facilities in Bukanga sub-county of Luuka District in October 2023 (the facilities from which participants in the pilot healthcare worker training came). Each pair of trainee facilitators included one youth researcher with a disability and one peer without a disability. One trainee was Deaf, one lived with albinism, and one had visual impairment. The trainee facilitator pairs visited facilities and completed the data collection form together with the healthcare worker, first taking relevant measurements before observing facility features and asking healthcare workers to report on activities and support available.

Outputs from the pilot will be used to inform further adaptations of the tool or protocol prior to full implementation across the remaining 38 primary health facilities in Luuka District later in 2024.

### Data analysis

Results from the pilot accessibility assessments were exported from REDCap and analysed in R using the *tidyverse*, *ggplot2*, *ggpubr* and *osmr* packages [30]. The number and percent of facilities meeting the criteria for each indicator was calculated. Summary scores for each sub-domain, domain and overall were generated by totalling the number of indicators in each group for which criteria had been met, divided by the number of indicators in the group and multiplied by 100. Scores are presented as the median and range across the pilot facilities. Radar charts and lollipop graphs were generated to visualise facility-level outputs.

Data from the feedback forms was manually extracted into an excel spreadsheet. Each open-text suggested solution was categorised into one of ten categories and a broad presumed investment level based on our knowledge of disability inclusive healthcare financing (Table 2), before being imported into R and summarised.

**Table 2:** Categorisation of suggested solutions in facility feedback forms

Categorisation	Example	Presumed Investment level
Developing new protocols and tools	Designing new impairment screening tool	Medium cost
Lobbying and engaging with policymakers	Lobbying District officials to provide health information in accessible formats	Low cost
Establishing a new role/creating a focal person among existing personnel	Designating a facility disability focal person	Low cost
Provision of alternative accessible formats for existing info	Developing Braille, pictorial versions and large print or easy read educational materials	Medium cost
Purchasing of new equipment	Purchasing an adjustable examination bed, handrails or tactile markers to indicate pathways	High, medium or low cost depending on type of equipment
Creation of new signage	Designating one latrine for persons with disabilities and clearly marking this with accessible signage	Low cost
Structural (major)	Building a ramp at the entrance	High cost
Structural (minor)	Levelling a path by filling in holes	Medium cost
Tidying	Removing boxes narrowing the useable width of a doorway	Low cost
Training	Ensuring at least one member of staff knows basic sign language	High cost

### Ethical approval

Ethical approval was granted by the London School of Hygiene & Tropical Medicine Observational Research Ethics Committee (LSHTM Ref 28327-1) and Uganda Virus Research Institute Research and Ethics Committee (UVRI REC Ref: GC/127/904) and Uganda National Council for Science and Technology (UNCST Ref: SS1348ES). Written informed consent was obtained from all participants. The study was conducted in accordance with the Declaration of Helsinki.

## Results

Five facilities (3 HC level II, and 2 HC level III) participated in the pilot accessibility assessments (Table 3). A healthcare worker who had recently undertaken the associated pilot healthcare worker training supported the assessment in each. The median completion time per assessment was 127 minutes (range 82–185), with few questions (2%) marked “not sure” by facilitators. Of these, explanatory notes captured in the data collection form suggested that the majority of instances where facilitators were unsure could be resolved through additional guidance on completion of the questions – for example defining a ”mainly flat” path in Question 2.1.

The nearest motorcycle taxi stage to the facility ranged from directly outside the facility to 2 km (approximately 26 minutes’ walk) away. The nearest taxi-bus route to the facility ranged from directly outside the facility to 6 km (over an hour’s walk) away.

**Table 3:** Information about the facilities, process of using the tool, and transportation in the area

Facility Information		*N* facilities	% facilities
Facility level	HC II	3	60
	HC III	2	40
Facility Type	Government	3	60
	Private (not for profit)	2	40
Health worker supporting assessment	None available	0	-
	Nurse	2	40
	Midwife	1	20
	Medical clinical officer	1	20
	Medical Records officer	1	20
Supporting Healthcare workers had received training on disability	Yes	5	100
	No	0	-
Process Information			Range
Duration	Time taken to complete audit (median mins)	107	82–185
Ease of completion	% items marked not sure	2	0–2.1
Transportation information		Median	Range
Bodaboda (motorcycle taxi)	Nearest boda stage (km)	0.16	0.01–1.94
	Travel time on foot (mins)	2.19	0.16–25.8
Taxi bus	Nearest taxi bus route (km)	0.04	0.02–5.24
	Travel time on foot (mins)	0.52	0.2–69.8

Median accessibility scores across the pilot facilities overall, by domain and sub-domain are presented in Table 4. Each category was equally weighted and median scores were calculated. The median overall facility accessibility score was 17.8% (range 12.3–28.8). Facilities performed best in the universal design and accessibility domain (25.8%, 22.6–41.9), followed by reasonable accommodation (20.0%, 6.7–33.3). Within universal design and accessibility, facilities scored better at entrances (42.8%, 14.3–57.1) and reception, corridors and waiting rooms (44.4%, 22.2–55.6) and lower for examination rooms and emergency routes (20.0%, 20.0–30.0) and toilets (0.0%, 0–40.0). Within reasonable accommodation, facilities scored similarly for both the information and communication (14.3%, 14.3–42.9) and assistance and support (25.0%, 0–25.0) sub-domains. Median scores for the capacity of facility staff domain (to identify and support people with disabilities) were low (6.67%, 6.7–20.0), with all 5 facilities scoring 0 for the sub-domains of specialist disability training of at least one staff member on duty and at least some healthcare workers. Median scores for linkages to other services were also low (0.0%, 0–25.0), with all 5 facilities scoring 0 for annually updated referral services, on median scoring 0% (0–50.0) for the availability of process data on disability, and 0.0% (0–33.3) for direct service linkages.

Indicator-level results for the 5 pilot facilities are summarised in Supplementary Tables 2–5.

Broadly, simple opportunities to meet accessibility standards were often missed. All 5 facilities provided parking, but none designated a space for patients with disabilities. All 5 had named receptions or triage areas but none had accessible signage at the entrance, in key areas or at the toilets. No pilot facilities had doors meeting access standards, height-adjustable examination beds, accessible emergency evacuation routes, assistive devices for temporary use, toilets meeting access standards or alternative formats for health information or general communication. Minimum criteria were often missed even where accessible features had been installed. For example, of three facilities with ramps, all reached the minimum required width and had textured, non-slip floors but two were too steep and none had handrails, meaning all failed the overall accessibility criteria for a ramp. One facility had a recently appointed disability focal person, but otherwise no disability-related training had been undertaken by any cadres in any facility (excluding the accompanying pilot training programme). One facility captured disability status in a register and used a referral and follow up monitoring tool, but none had screening tools or routine summaries of patients with disabilities served, had annuallyupdated referral processes for relevant services or had direct links with organisations of people with disabilities (OPDs) or rehabilitation outreach.

**Table 4:** Median accessibility scores overall, by domain and by sub-domain across facilities

		Median (Max 100)	Range
Domain	Sub-Domain		
Universal Design and Accessibility	Entrance to services	42.8	14.3–57.1
	Reception, corridors and waiting rooms	44.4	22.2–55.6
	Examination rooms and emergency routes	20.0	20.0–30.0
	Toilets	0	0–40.0
	Domain total	25.8	22.6–41.9
Reasonable accommodation of people with disabilities	Information and communication	14.3	14.3–42.9
	Assistance and support	25.0	0–25.0
	Domain total	20.0	6.7–33.3
Capacity of facility staff to identify and support people with disabilities	Awareness and accessibility training (all)	0	0–50.0
	Specialist training for at least one staff member on duty	0	0
	Healthcare worker training (at least some staff)	0	0
	Other training and support capacity	20	20.0–20 0.0
	Domain total	6.67	6.7–20.0
Linkage to disability and rehabilitation services	Disability process data	0	0–50.0
	Annually updated referral processes	0	0
	Direct service linkages	0	0–33.3
	Domain total	0	0–25.0
**Total Score**		**17.8**	**12.3–28.8**

**Fig. 1:**
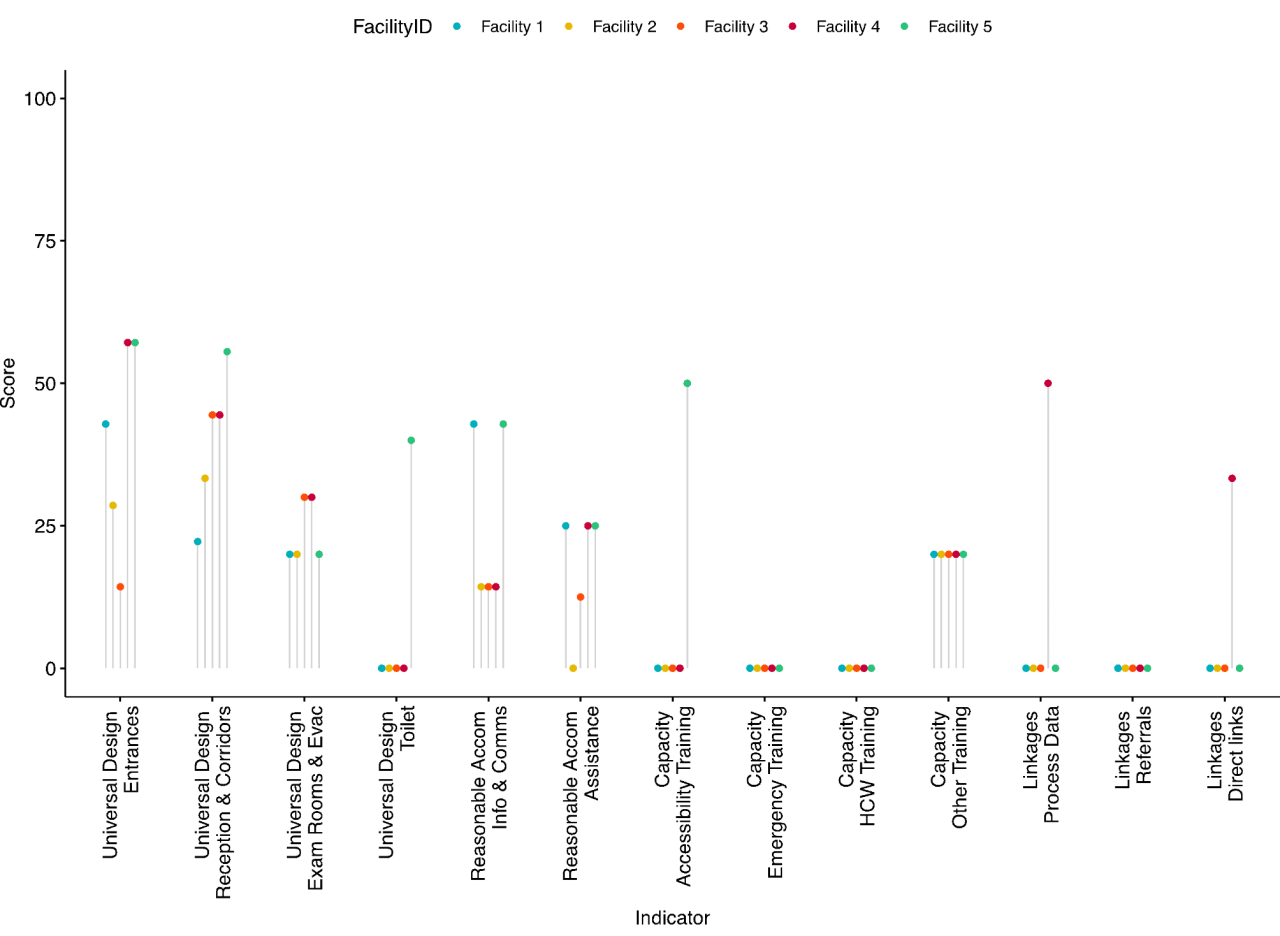
Facility-level sub-domain summary scores (out of 100) by indicator and facility

Facility-level data for each sub-domain is shown in Fig. 1, with underlying data and additional facility radar charts tabulated in Supplementary File Table 6; Fig. 1. Facilities 4 and 5 scored highest overall and across the 4 domains, driven by comparatively higher scores for linkages (Facility 4, 25.0%), and universal design and reasonable accommodation (Facility 5, 41.9% and 33.3% respectively). In particular, Facility 4 scored highest in the sub-domains of collecting disability process data (50%) and having direct links with onward services (33%), and Facility 5 in the sub-domains of universal design: toilet (40.0%) and reasonable accommodation: information and communication (42.9%).

A median of 21 suggestions (range 14–26) were made for each facility in the feedback forms completed at the end of the assessment. The most common suggestions fell under the category of minor structural changes (20% of suggestions) – for example smoothing pathways or constructing short ramps in doorways and entrances. Other common suggestions fell under the categories of developing new tools and protocols (11% of suggestions), improving signage (11%), training opportunities (11%) and lobbying and engaging with decision makers (10%). Around a quarter (26%) of suggestions were considered to incur a high cost, 40% medium, 2% low and 33% low cost (See Table 5).

**Table 5:** Feedback form suggestion summary

	Count	
Categorisation	*N*	%
Developing new protocols and tools	12	11.3
Lobbying and engaging with policymakers	11	10.4
Establishing a new role/creating a focal person among existing personnel	4	3.8
Provision of alternative accessible formats for existing info	8	7.5
Purchasing of new equipment	9	8.5
Creation of new signage	12	11.3
Structural (major)	9	8.5
Structural (minor)	21	19.8
Tidying	8	7.5
Training	12	11.3
**Investment Level**		
High	27	25.5
Medium	42	39.6
Low	2	1.9
No cost	35	33.0

## Discussion

The aim of this study was to adapt and pilot a tool to assess the accessibility of primary health facilities in Luuka District, Uganda. Overall, the five facilities participating in our pilot scored low in our accessibility assessment (median total score 17.8%), with lowest scores for the domains of staff capacity to identify and support people with disabilities (6.67%) and linkages to disability and rehabilitation services (0.0%). There is no other published data using the DAC with which to compare our findings. However, findings from health facility assessments in Brazil, India and Bangladesh using similar tools mirror ours – for example an average 18.7% overall accessibility score across nearly 40,000 primary health centres in Brazil, and an average 15.7% score for disability-friendly health care in 150 primary to tertiary public health facilities in Bangladesh [17–19]. Moreover, these studies also show a trend towards lower scores for information/communication and capacity of healthcare providers, compared with scores for physical accessibility and infrastructure. For example, a census of 67 primary health centres across one district of India estimated that while the majority had accessible entrances and routes through the facilities (both 85.1%), only 7.5% had accessible signage [18]. These findings suggest that the adapted DAC performed as expected compared with similar tools used in both middle- and low-income settings and is appropriate for future use in Uganda.

In our pilot, we estimated the median distance from facilities to the nearest taxi stage and public transport drop off zone. Geographical accessibility of health facilities as a metric of health care equity has received considerably more attention than accessibility for people with disabilities in Uganda and elsewhere in the region [31, 32]. However, these studies tend to focus on distances by useable road between patients’ homes and facilities only. Our findings highlight the additional distances patients may have to cover on foot from public drop off zones. Future geographical and disability accessibility studies should be triangulated with patient data to account for the mode of transportation taken by patients, the ensuing distance travelled on foot, and any functional limitations that may make this more challenging for some patients.

Our pilot findings and those from elsewhere confirm that there is substantial value in conceptualising access to health services more broadly than physical accessibility, and that tools such as the DAC to measure this at the facility level can provide valuable additional insights. This is also in keeping with recent recommendations from the World Bank, the World Health Organization and other sources, in which progress towards disability-inclusive health systems is conceptualised not only as physically accessible services, but also accessible health information and communication, an appropriately trained health workforce and quality, and the provision of affordable healthcare for all [5, 11, 33]. While this necessarily requires system-level resourcing and governance, facility-level data can provide valuable complementary understanding of the current status of disability-inclusion, to support policy and programme change. Our pilot study was too small to analyse patterns or predictors of higher or lower disability-inclusion between facilities, but this could be generated from larger datasets to prioritise facilities for improvement or identify facilitators for success.

Over a third of the suggested facility improvements arrived at in the pilot between the DAC facilitators and healthcare workers were no or low cost, highlighting the simple and cost-effective ways that individual facilities and their workforces can improve their service provision in tandem with health-system level developments. Empowering healthcare workers with simple, cost-effective facility improvements not only benefits individual facilities but also complements broader health-system level advancements, promising more efficient and accessible healthcare services. Involving persons with disabilities as facilitators of accessibility assessments also showed promising results. The youth researchers were highly motivated and dynamic in their roles, using their personal experiences to contextualise the findings with their patient perspective, and identify simple solutions together with healthcare workers in the spirit of collective responsibility. In this co-development process, they were able to leverage their respective expertise and experiences to ensure that proposed improvements were both practical and meaningful. This participatory approach not only enhanced the feasibility of implementing suggested changes by also fostered a sense of ownership among all stakeholders involved [34–36].

To better reflect local conditions, our team made several modifications to the DAC, resulting in 20% more indicators than the original DAC. Our adapted version of the DAC took between one and three hours to complete. These adaptations were necessary to ensure the tool’s relevance and comprehensiveness in the study context. For detailed information on the specific changes made, including the additional response options and the full list of adapted indicators, please refer to the supplementary materials (see Appendix 2).

While it is anticipated that length of completion may diminish over time as facilitators become more experienced, the increase in indicators is indicative of the complexities of inclusion requirements from the perspectives of patients with disabilities, and the heterogeneity of these. Indeed, there are further indicators that were not assessed in the adapted tool, such as lighting, which may impact how people with visual or developmental disabilities access health facilities [37, 38]. There is an ongoing trade-off between completeness and feasibility, which led us to prioritise the most critical indicators for health service access. We will embed a ranking exercise in upcoming data collection with patients with disabilities as part of our broader study, to inform future adaptations. This will also allow us to assess whether any weighting of domains or sub-domains is beneficial. Very few questions (2%) were marked difficult to answer by facilitators participating in the pilot, suggesting few further adaptations are required for the full implementation. However, adaptation may be necessary for other contexts, as local accessibility laws vary by country [39], and so local adaptation should be considered a critical step in any future work around prioritisation or weighting.

Our study had several strengths. We conducted the first accessibility assessment of primary health facilities in Uganda, using an adapted version of a validated tool deployed by facilitators with disabilities. Our adaptations were developed in consultation with Ugandan health service-users with disabilities and in line with local accessibility standards, maximising the face validity and context relevance of the assessment. We recognize that the involvement of only young researchers with disabilities may have influenced our findings. The perspectives of older individuals with disabilities were not represented in the research team, and future studies would benefit from including researchers of varying ages to capture a more diverse range of experiences and insights, particularly from the elderly population. Additionally, our pilot sample was small, limiting generalisability of the findings or ability to conduct some analyses. We intend to complete assessments in the remaining 38 primary health facilities in Luuka in July – September 2024 to address these limitations.

### Conclusion

We pilot-tested an adapted version of the Disability Awareness Checklist in 5 primary health facilities in Luuka District, Uganda. We found that overall accessibility scores in the pilot facilities were low, with many opportunities for low-cost improvement at the facility level. We did not identify any issues with the implementation of our adapted version of the tool, suggesting few further adaptations are required for its future use in this setting.

The authors express their gratitude to the DAC facilitators who undertook the pilot accessibility assessments and the healthcare workers who accompanied them at the pilot facilities, and to the remaining members of the Disabled Youth Investigator Group who participated in the DAC adaptation and training exercises (Naume Adong, Betty Akwii, Aisha Nalikka, Richard Luzinda).

HK conceptualised the study. IZM, ASS, FBM and HK developed the methodology for the study design. IZM, ASS, FBM and AM developed and led the training for the data collection. ASS and AM oversaw data collection activities. IZM, ASS, TS and SR developed the data analysis plan. IZM completed the analyses. IZM drafted the manuscript. ASS, AM, TS, SR, SM, FBM and HK reviewed and revised the manuscript. All authors read and approved the final manuscript.

This project is funded by the National Institute for Health Research (NIHR) [NIHR Global Research Professorship (Grant Reference Number NIHR301621)] awarded to Prof. Hannah Kuper. The views expressed in this publication are those of the author(s) and not necessarily those of the NIHR.

The datasets generated and/or analysed during the current study are not publicly available due to the small size of the dataset and potential for identification of individual health centres. Data may be available from the corresponding author on reasonable request.

## Electronic supplementary material

Below is the link to the electronic supplementary material.

Supplementary Material 1

## Data Availability

Data will be provided upon written request to the authors.

## Abbreviations

AS: Andrew Sentoogo Ssemata
AM: Abdmagidu Menya
DAC: Disability Awareness Checklist
DYI: Disabled Youth Investigates Research programme
HC II: Health Centre II
HC III: Health Centre III
HC IV: Health Centre IV
IZM: Islay Mactaggart
LMICs: Low-and middle-income countries
LSHTM: London School of Hygiene & Tropical Medicine
MRC/UVRI: Medical Research Council/Uganda Virus Research Institute
