# Supplementary material for: Adapting and pilot testing a tool to assess the accessibility of primary health facilities for people with disabilities in Luuka District, Uganda
Source: Int J Equity Health. 2024 Nov 13;23:237. doi: 10.1186/s12939-024-02314-0 (PMC11562328; doi:10.1186/s12939-024-02314-0)
Supplement: Supplementary file 1 — Supplementary Material 1 [file 12939_2024_2314_MOESM1_ESM.docx]

# **Adapting and pilot testing a tool to assess the disability-inclusivity of primary health facilities for people with disabilities in Luuka District, Uganda**

Table of Contents

[Supplementary Tables and Figures 1](#_Toc175125458)

[Appendix 1: Accessibility Standards Minimum Criteria 13](#_Toc175125459)

[Appendix 2: Adapted DAC used in pilot 15](#_Toc175125460)

[Appendix 3: Disability Access checklist facility feedback template 23](#_Toc175125461)

**List of Tables and Figures**

[*Table 1: Summary of Uganda’s primary Health System 2*](#_Toc175125780)

[*Table 2: Number and proportion of facilities meeting criteria for individual indicators in the Universal Design and Accessibility Domain 2*](#_Toc175125781)

[*Table 3: Number and proportion of facilities meeting criteria for individual indicators in the Reasonable Accommodation of people with disabilities domain 3*](#_Toc175125782)

[*Table 4: Number and proportion of facilities meeting criteria for individual indicators in the Capacity of facility staff to identify and support people with disabilities domain 4*](#_Toc175125783)

[*Table 5: Number and proportion of facilities meeting criteria for individual indicators in the Linkages to disability and rehabilitation services domain 5*](#_Toc175125784)

[*Table 6: Facility-level domain and sub-domain scores (underlying data for graphs 1 and 2) 6*](#_Toc175125785)

[*Table 7: Summary of Rapid Review of Disability Audit Tools 7*](#_Toc175125786)

[*Figure 1: Facility-level domain summary scores 12*](#_Toc163658298)

## Supplementary Tables and Figures

Table 1: Summary of Uganda’s primary Health System

| Type | Level | Coverage (approx.) | Minimum staffing | Care | No. in Luuka |
| --- | --- | --- | --- | --- | --- |
| VHT/ HCI | Village | 4 per village | Volunteer community members with basic training | Recording demographic and health data, health education and mobilisation | 1275 |
| HC II | Parish or ward | 5,000 | One nurse, one midwife and two nursing assistants | Outpatient, antenatal, immunization and outreach | 30 |
| HC III | Sub-county or division | 20,000 | As above, plus one clinical officer, additional midwife, health assistant, records officer and lab assistant | As above, plus maternity, inpatient and laboratory services | 12 |
| HC IV | County or municipality | 100,000 | As above, plus one medical officer, additional nurses, clinical officers and midwives, a health inspector and dispenser | As above, plus emergency surgery and blood transfusion services | 1 |
| Table adapted from data in Turyamureba et al. 2023 and Mays et al. 2017 (1,2) | | | | | |

Table 2: Number and proportion of facilities meeting criteria for individual indicators in the Universal Design and Accessibility Domain

| Indicator | N facilities | %  facilities |
| --- | --- | --- |
| Entrance to services |  |  |
| Path to main entrance is mainly smooth and flat | 2 | 40 |
| Facility has space to park a car | 5 | 100 |
| Facility has accessible parking space | 0 | 0 |
| Facility has a ramp | 3 | 60 |
| - Ramp meets access standards^1^ | 0 | 0 |
| Main facility entrance is accessible^2^ | 3 | 60 |
| Facility has accessible signage | 1 | 20 |
| Reception, corridors and waiting rooms |  |  |
| Facility has named reception or triage area | 5 | 100 |
| - Named reception or triage area is accessible^3^ | 2 | 40 |
| - Reception or triage desk staff member is accessible | 4 | 80 |
| - Reception or triage desk meets access standards (height <800mm) | 3 | 60 |
| Corridor and room floors are non-slip | 1 | 20 |
| Facility doors (e.g. office, pharmacy) meet access standards (width>800mm) | 0 | 0 |
| Facility corridors meet access standards (width>800mm) | 3 | 60 |
| Directions to key areas are signposted | 0 | 0 |
| Directions to key areas are signposted in accessible formats | - | - |
| Examination Rooms and emergency evacuations |  |  |
| Examination room entrance meets access standards (width>800mm) | 1 | 20 |
| All service rooms are on the same level | 5 | 100 |
| - If not, functional lift or ramp to upper floors exists | - | - |
| A height adjustable examination table is available | 0 | 0 |
| A height adjustable bed is available | 1 | 20 |
| Caretakers/support persons are welcomed and allowed to support people with disabilities in the process of accessing care | 5 | 100 |
| Emergency evacuation route exists | 0 | 0 |
| - Emergency evacuation route is wheelchair accessible | - | - |
| - Emergency evacuation route is signed with pictures and Braille | - | - |
| - Emergency evacuation route is fitted with warning lights for d/Deaf or hearing impaired | - | - |
| Toilets |  |  |
| Facility has a designated disability accessible toilet or latrine | 1 | 20 |
| - Toilet/latrine is labelled as disability accessible | 0 | 0 |
| - Toilet/latrine meets access standards (width>900mm) | 0 | 0 |
| - Toilet/latrine has washpoints that a wheelchair user can reach | 1 | 20 |
| - Toilet/latrine has handrails | 0 | 0 |
| ^1^Composite score if ramp slope (min 1:12) and width (min 1300mm) is acceptable, has handrails and is non-slip  ^2^Composite score based on useable width (min 900mm) and weight of entrance door  ^3^Composite score based on reception being named, attended, clearly marked and near the main entrance | | |

Table 3: Number and proportion of facilities meeting criteria for individual indicators in the Reasonable Accommodation of people with disabilities domain

| Indicator | N facilities | %  facilities |
| --- | --- | --- |
| Information and communication |  |  |
| Facility has alternative communication techniques for people who require them | 0 | 0 |
| Facility provides written health information for patients (e.g. vaccination leaflet) | 1 | 20 |
| - Health information is available in alternative formats | 0 | 0 |
| Facility provides written informed consent sheets for patients | 2 | 40 |
| - Informed consent is available in alternative formats | 0 | 0 |
| Facility provides information on medicines and where to access them to patients | 5 | 100 |
| - Facility provides information about more convenient dispensary options available to people with disabilities | 1 | 20 |
| Assistance and support |  |  |
| Assistance is available to fill in forms or documents as needed | 3 | 60 |
| Functional assistive devices are available for temporary use | 0 | 0 |
| Guiding services are available if required | 2 | 40 |
| Facility has a mechanism for people to make complaints or suggestions | 2 | 40 |
| - Complaints or suggestions mechanism is available in alternative formats | 0 | 0 |
| Facility undertakes routine patient satisfaction surveys | 0 | 0 |
| - Routine satisfaction surveys are available in alternative formats | 0 | 0 |
| There is clear signage indicating that people with disabilities will be moved to the front of the queue if required | 0 | 0 |

Table 4: Number and proportion of facilities meeting criteria for individual indicators in the Capacity of facility staff to identify and support people with disabilities domain

| Indicator | N facilities | % of  Facilities reporting (/5) |
| --- | --- | --- |
| Awareness and accessibility training |  |  |
| Facility has disability focal person | 1 | 20 |
| - Facility focal person has been provided with training in supporting patients with disabilities | 1 | 20 |
| At least some staff members have been trained in disability accessibility issues^*^ | 0 | 0 |
| All staff in all cadres have been trained on disability etiquette or sensitisation (including accessibility) | 0 | 0 |
| Specialist Training for at least one staff member on duty |  |  |
| Supporting emergency evacuation for people of disabilities | 0 | 0 |
| Basic sign language interpretation and Braille signage | 0 | 0 |
| Training on disability-related violence | 0 | 0 |
| Healthcare worker training (at least some staff) |  |  |
| How to screen for and identify disability | 0 | 0 |
| How to refer for rehabilitation/specialized hospital services e.g. physiotherapy or occupational therapy? | 0 | 0 |
| Awareness and use of assistive devices e.g. wheelchairs, white cane? | 0 | 0 |
| Other training and support capacity |  |  |
| At least some staff members have been trained in Sexual Reproductive Health and Rights (SRHR) and gender-based violence (GBV)? | 0 | 0 |
| - This training included people with disabilities | 0 | 0 |
| Service protocols or guidelines incorporate specific guidance on addressing the needs of people with disability | 0 | 0 |
| Health promotional and educational materials are provided by the facility | 5 | 100 |
| - These materials are available in alternative formats | 0 | 0 |
| *Excludes staff trained in accompanying pilot healthcare worker training programme | | |

Table 5: Number and proportion of facilities meeting criteria for individual indicators in the Linkages to disability and rehabilitation services domain

| Indicator | N facilities | %  facilities |
| --- | --- | --- |
| Disability process data |  |  |
| Registers that capture disability status and needed accommodations exist | 1 | 20 |
| Screening tools are available and used to identify impairments in children and adults (e.g. physical, mental, visual, hearing, intellectual) | 0 | 0 |
| Routine data summaries include people with disabilities served by the facility | 0 | 0 |
| A referral and follow up monitoring tool exists | 1 | 20 |
| Annually updated referral processes for the following exist |  |  |
| Disability services providing assistive devices | 0 | 0 |
| Referral and follow-up system for repairing assistive devices | 0 | 0 |
| Rehabilitation/specialized medical services | 0 | 0 |
| Mental healthcare providers/services accessible to people with disabilities | 0 | 0 |
| Directory of organizations for people with disabilities available at the facility for ease of access to additional support services | 0 | 0 |
| Direct service linkages |  |  |
| Linkages and active collaborations with organisations for people with disabilities exist | 0 | 0 |
| Linkages with a rehabilitation outreach programme that visits the facility at least every 6 months | 0 | 0 |
| linkages and referral to Home-Based or Community- Based Rehabilitation programmes in the area | 1 | 20 |

Table 6: Facility-level domain and sub-domain scores (underlying data for graphs 1 and 2)

|  |  | Facility Anonymised ID | | | | |
| --- | --- | --- | --- | --- | --- | --- |
|  |  | 1 | 2 | 3 | 4 | 5 |
| Domain | Sub-Domain |  |  |  |  |  |
| Universal Design and Accessibility | Entrance to services | 42.9 | 28.6 | 14.3 | 57.1 | 57.1 |
|  | Reception, corridors and waiting rooms | 22.2 | 33.3 | 44.4 | 44.4 | 55.6 |
|  | Examination rooms and emergency routes | 20.0 | 20.0 | 30.0 | 30.0 | 20.0 |
|  | Toilets | 0 | 0 | 0 | 0 | 40.0 |
|  | Domain total | 22.6 | 22.6 | 25.8 | 35.5 | 41.9 |
| Reasonable accommodation of people with disabilities | Information and communication | 42.9 | 14.3 | 14.3 | 14.3 | 42.9 |
|  | Assistance and support | 25.0 | 0 | 12.5 | 25.0 | 25.0 |
|  | Domain total | 33.3 | 6.7 | 13.3 | 20.0 | 33.3 |
| Capacity of facility staff to identify and support people with disabilities | Awareness and accessibility training (all) | 0 | 0 | 0 | 0 | 50.0 |
|  | Specialist training for at least one staff member on duty | 0 | 0 | 0 | 0 | 0 |
|  | Healthcare worker training (at least some staff) | 0 | 0 | 0 | 0 | 0 |
|  | Other training and support capacity | 20.0 | 20.0 | 20.0 | 20.0 | 20.0 |
|  | Domain total | 6.7 | 6.7 | 6.7 | 6.7 | 20.0 |
| Linkage to disability and rehabilitation services | Disability process data | 0 | 0 | 0 | 50.0 | 0 |
|  | Annually updated referral processes | 0 | 0 | 0 | 0 | 0 |
|  | Direct service linkages | 0 | 0 | 0 | 33.3 | 0 |
|  | Domain total | 0 | 0 | 0 | 25.0 | 0 |
| **Total Score** | | 17.8 | 12.3 | 15.1 | 24.7 | 28.8 |

| Table 7: Summary of Rapid Review of Disability Audit Tools | | | | | | | | | |
| --- | --- | --- | --- | --- | --- | --- | --- | --- | --- |
| Tool Name | Citation | Tool Type | Framework/ Development background | Domains included | # of questions and Response options | Scoring Methodology | Auditor Training Documentation | Where used/tested | Results of use/testing |
| Disability Awareness Checklist | Hanass-Hancock J, Willan S, Carpenter B, Tesfay N, Dunkle K. Disability Awareness Checklist for health care services [Internet]. Durban, South Africa: South African Medical Research Council; 2023. Available from: https://www.samrc.ac.za/intramural-research-units/testing-gbv-quality-assurance-tool | Structured Questionnaire and observational checklist | Based on principles/ concepts of UN CRPD and informed by existing disability audits. Design completed via comprehensive expert review in 2021 | universal design and accessibility; reasonable accommodation; capacity of facility staff to identify and support people with disabilities; and linkages to disability and rehabilitation services | 5 sub-domains and 59 indicators; Yes/ No/ Not sure | Binary 1/0 per question transformed into 0-100 score per domain and overall. All sub scales attributed same weight in overall score. Access to automated analyses via redcap | Facilitators guide and training presentations | South Africa | Not published, but pilot tested during a study focusing on the adaptation of the WHO post-Gender-based Violence Quality Assurance tool (GBV-QA tool) in 2022 |
| Sightsavers Accessibility Audit | Pregel A, Smith, K. and Bridger, K: **Accessibility standards and audit pack**. In*.* Edited by Sightsavers. Haywards Heath; 2019. | Checklist against accessibility standards develooped as part of package | UN CRPD, international accessibility standards and principles of universal design | Access to the facility, Entrance, reception and waiting area, Rooms and halls, Circulation paths and internal wayfinding, General toilets, Accessible toilets, Accessible showers, Lifts and wheelchair platform stairlifts | 174; Yes/ No/ NA | Unclear, but overall score (and per section) out of 100 categorised as minimal accessibility (0-25), low (26-50), medium (51-75) and high (76-100). | In depth. Includes 2 day training course (of which 1 is practical exercises) | Unclear/ no published results or centralised repository. Blog suggests use in Ghana and Nigeria (see below), assume others too. | Not published |
| Sightsavers Accessibility Audit = humanitarian settings | As above | As above | As above | As above | As above | As above | Three two-hour online training sessions over two days with practical assignments offline | Nigeria 2021 | Learning report from Nigeria 2021 suggests SS looking to adapt for Humanitarian settings |
| Disability Friendly Health Services Checklist | Torsha N, Rahman FN, Hossain MS, Chowdhury HA, Kim M, Rahman SMM, Rahman A, Rahman A: Disability-friendly healthcare at public health facilities in Bangladesh: a mixed-method study to explore the existing situation. BMC Health Serv Res 2022, 22(1):1178. | Observational Checklist | Draws on Ireland Natonal Guidelines on Accessible health and Social Care Services, and UN Disability-Inclusive Health Services in Africa Toolkit. Groups into domains based on framework developed by authors. | Information, Communication, Infrastructure and Capacity Development | Infrastructure Indicators with sub-points  30 readiness indicators for each of Information, Communication, Infrastructure and Capacity Development;  Yes/No | Infrastructure: Each sub-point scored 1 if yes to give score per indicator plus composite scores per facility, and mean scores for indicators and facilities across 150 surveyed  Readiness: Domain specific score (sum of indicators per domain/number indicators per domain) | Not publically available | Used Bangladesh, no pilot testing described | Entrances and consulting rooms most accessible, wards, outpatient departments and ticket counters least accessible |
| External Evaluation of the Brazilian National Program for Improving Access to Primary Care–PMAQ-AB | Pinto A, Köptcke LS, David R, Kuper H. A National Accessibility Audit of Primary Health Care Facilities in Brazil-Are People with Disabilities Being Denied Their Right to Health? Int J Environ Res Public Health. 2021 Mar 13;18(6):2953. doi: 10.3390/ijerph18062953. PMID: 33805773; PMCID: PMC7999795. | Structured Questionnaire and observational checklist | Census of PHC Facilities within MoH funded program for evaluating access to primary care (PMAQ-AB). | Internal and External building accessibility, Information accessibility and availability of healthcare personnel trained in disability-related issues | 22; Yes/No | Binary 1/0 per question. Three sub scales for Internal Access, External Access and Information Access transformed into 0-100 score. Plus overall score also transformed into 0-100. All three sub scales attributed same weight in overall score. | Not publically available | Developed and used Brazil | Low overall accessibility and by sub-domain |
| Physical Accessibility Audit Checklist developed by Accessible India Campaign | Nischith, K. R., M. Bhargava, and K. M. Akshaya. "Physical accessibility audit of primary health centers for people with disabilities: An on-site assessment from Dakshina Kannada district in Southern India." *Journal of Family Medicine and Primary Care* 7.6 (2018): 1300-1303. | Observational Checklist | Developed by Accessible India Campaign, paper describes national law and UNCRPD but doesn’t explictly state how checklist was developed | parking and exterior access, accessible entrance and internal environment, doors and doorways, and accessible toilet | Parking and Exterior Access 24 indicators; Accessible entrance 31 indicators, doors and doorways and accessible toilet not described, 116 questions total;  Yes/No | Not provided, paper reports % of facilities achieving specific indicator | Describes a handbook not clearly available online | One district in India | Some indicators zero % across all facilities, range of other estimates |
| universal access comparison guideline in PHC centers. | Campillay-Campillay M, Calle-Carrasco A, Dubo P, Moraga-Rodríguez J, Coss-Mandiola J, Vanegas-López J, Rojas A, Carrasco R. Accessibility in People with Disabilities in Primary Healthcare Centers: A Dimension of the Quality of Care. Int J Environ Res Public Health. 2022 Sep 29;19(19):12439. doi: 10.3390/ijerph191912439. PMID: 36231740; PMCID: PMC9564706. | Observational Checklist | National Technical Standard based on Spanish Technical Standard for Accessibility, complemented by liteerature search on universal accessibility criteria | (i) participation, (ii) information, (iii) accessibility chain and (iv) other architectural aspects. | he techniques of observation and verification of compliance with technical criteria were used: taking measurements using a tape measure, simulating a person with a disability using a wheelchair to make the routes, checking flows and spaces in situ, photographs of plans and checking compliance with standards, following the access chain from the transport stops public to the health center. | Not described | Not publically available | 18 Primary Health Centres in Atacama region, Chile | Very low compliance averages were obtained, 37.7% participation, 4% information, 44.4% access chain, and 63.9% architectural aspects, |

Figure 1: Facility-level domain summary scores

## Appendix 1: Accessibility Standards Minimum Criteria

| **DAC Key Measurements of the Facilities** | **UNAPD Accessibility Standards 2010 (3)** | **The Building Control (Accessibility Standards For Persons With Disabilities) Code, 2019 (4)** |
| --- | --- | --- |
| 1.1 Distance from the nearest public transportation pick-up/drop off point to the facility entrance. | Not described | Not described |
| 1.2 Degree of slope of ramp (if any) | 1.3 The maximum recommended slope of ramps is 1:20 (5 cm pr. m) (See fig. 1.3). For small level dif- ferences and very short ramps of about 1 meter, the maximum slope of such a ramp should be 1:10 (10 cm pr. m). | 12 (3a) A ramp provided under this Code shall— (a) have a gradient measured along the centre line, that is not steeper than 1:12 as prescribed in Figure 10 in the Schedule; (e) have a handrail on both sides of the ramp or, where the width is greater than two thousand four hundred millimetres, an immediate handrail at one thousand, one hundred millimetres in accordance with paragraph 16 on the run; |
| 1.3 Usable width of main facility entrance door (measure at the narrowest point, accounting for security gate, furniture, or other barriers) | 3.4 The minimum opening space of entrance when the door is fully opened should be 0.90m. | 10 (2) A clear opening shall be at least nine hundred millimetres when approached along a line that is perpendicular to the opening as is prescribed in Figure 6, Figure 7, Figure 8 and Figure 9 in the Schedule. |
| 1.4 Usable width of doorways to examination rooms (measure at the narrowest point, accounting for security gate, furniture, or other barriers) | 4.2 The interior doors should have a minimum clear opening of 0.80m when the door is fully open. | FIGURE 8- DOORWAY AND DOOR (900mm) |
| 1.5 Usable width of toilet door width for the wheelchair accessible toilet (put 0 if no accessible toilet) | 4.2 The interior doors for persons with disability’s toilet should have a minimum clear opening of 0.90m | 16 (2) A door into an accessible toilet shall have a clear opening of at least nine hundred millimetres. |
| 1.6 Width of other facility doors that patients use e.g office, pharmacy (measure at the narrowest point, accounting for security gate, furniture, or other barriers) | 4.2 The interior doors should have a minimum clear opening of 0.80m when the door is fully open. | FIGURE 8- DOORWAY AND DOOR (900mm) |
| 1.7 Usable width of corridors (measure at the narrowest point, accounting for security gate, furniture, or other barriers) | 9.2 The minimum width of unobstructed pathway/ corridor should be 1.3m and preferably 1.5m to allow maneuvering of wheelchairs through lateral doors. | FIGURE 4- OBSTRUCTION IN PATH OF TRAVEL (1500mm) |
| 1.8 Height of reception desk as it faces patients | 16.6 The maximum height of an accessible counter should be 0.80m high. | Not described |

## Appendix 2: Adapted DAC used in pilot

| **DAC** | | |
| --- | --- | --- |
| **Background Information** | | |
| Question | Response Options |  |
| 1.0 Facility ID: | Open text field |  |
| 1.1 Facility Type: | ☐ Government  ☐ Private (not for profit)  ☐ Private |  |
| 1.2: Name of Health facility: | ☐ HC II  ☐ HC III  ☐ HC IV |  |
| 1.4a: Audit date: | Date input |  |
| 1.4b: Audit start time: | Time input |  |
| 1.5: Auditor Pair:  *Select the correct pair* |  |  |
| 1.6: Health worker available to accompany team for audit? | ☐ Yes  ☐ No |  |
| 1.7: If yes, profession of health worker | ☐ Medical Officer  ☐ Nurse  ☐ Clinical officer  ☐ Midwife  ☐ Laboratory Tech  ☐ Dentist  ☐ Health educator  ☐ Health assistant  ☐ Health inspector  Other |  |
| 1.7.1: If other profession, specify: | Open text field |  |
| **Universal Design and Accessibility – Entrance to Services** | | |
| 2.1 Is the path from the gate to the main entrance mainly flat and smooth? | ☐ Yes  ☐ No  ☐ Not sure |  |
| 2.1 Note: | Open text field |  |
| 2.2 Does the facility have space to park a car? | ☐ Yes  ☐ No |  |
| 2.2.1 If yes, is there a clearly signed parking space/s for people with disabilities with access pathway to the front door? | ☐ Yes  ☐ No  ☐ Not sure |  |
| 2.2.1 Note: | Open text field |  |
| 2.3 Does the facility have a ramp at at least one entrance | ☐ Yes  ☐ No |  |
| 2.3.1 If yes, width of ramp | *Measurement (cm)* |  |
| 2.3.2 If yes, are hand rails present? | ☐ Yes  ☐ No |  |
| 2.3.3 If yes, texture of the ramp | ☐ Smooth concrete  ☐ Textured/ rough concrete  ☐ Smooth wood or planks  ☐ Textured/ rough wood or planks  ☐ Smooth metal  ☐ Textured/ rough metal  ☐ Smooth plastic  ☐ Textured/ rough tile  ☐ Other |  |
| 2.3.3.1 If other texture, specify | Open text field |  |
| 2.3.4 If yes, what is the degree of the slope of the ramp? | *Measurement (degrees)* |  |
| 2.3.5 If no, is there at least one entrance level to the outside floor? | ☐ Yes  ☐ No |  |
| 2.4 Usable width of main facility entrance door (measure at the narrowest point, accounting for security gate, furniture, or other barriers) | *Measurement (cm)* |  |
| 2.5 Does the facility entrance door open easily with one hand? | ☐ Yes  ☐ No  ☐ Not sure |  |
| 2.5 Note: | Open text field |  |
| 2.6 Does the facility have signage at the entrance? | ☐ Yes  ☐ No |  |
| 2.6.1 If yes, is this signage available in accessible formats e.g large print or braille? | ☐ Yes  ☐ No |  |
| **Universal design and accessibility - Reception, corridors and waiting rooms** | |  |
| 3.0 Does the facility have a named reception | ☐ Yes  ☐ No |  |
| 3.0.1 If yes, does reception have a regular full time staff attending to patients? | ☐ Yes  ☐ No |  |
| 3.0.2 If yes, is the reception clearly marked? | ☐ Yes  ☐ No |  |
| 3.0.3 If yes, is reception near the main entrance and accessible? | ☐ Yes  ☐ No |  |
| 3.0.4 If yes, are you able to easily communicate/ interact with receptionist? | ☐ Yes  ☐ No |  |
| 3.0a Does the facility have a first place you reach/triage? | ☐ Yes  ☐ No |  |
| 3.01a If yes to 3.0a, does the first place you reach/triage have a regular full time staff attending to patients? | ☐ Yes  ☐ No |  |
| 3.0.2 If yes to 3.0a, is the first place you reach/triage clearly marked? | ☐ Yes  ☐ No |  |
| 3.0.3 If yes to 3.0a, is the first place you reach/triage near the main entrance and accessible? | ☐ Yes  ☐ No |  |
| 3.0.4 If yes to 3.0a, are you able to easily communicate/ interact with the member of staff? | ☐ Yes  ☐ No |  |
| 3.0.5 If yes, what is the height of reception desk as it faces patients | *Measurement (cm)* |  |
| 3.0.6 If yes, what is the height of first point/triage desk as it faces patients | *Measurement (cm)* |  |
| 3.1 Usable width of doorways to examination rooms (measure at the narrowest point, accounting for security gate, furniture, or other barriers) | *Measurement (cm)* |  |
| 3.2 Width of other facility doors that patients use e.g office, pharmacy (measure at the narrowest point, accounting for security gate, furniture, or other barriers) | *Measurement (cm)* |  |
| 3.3 Usable width of corridors (measure at the narrowest point, accounting for security gate, furniture, or other barriers) | *Measurement (cm)* |  |
| 3.4 Are there directions in key areas? | ☐ Yes  ☐ No  ☐ Not sure |  |
| 3.4 Note: | Open text field |  |
| 3.4.1 If yes, are they in accessible formats like lifts, signposts? | ☐ Yes  ☐ No  ☐ Not sure |  |
| 3.4.1 Note: | Open text field |  |
| 3.5 Do emergency evacuation/exit routes exist? | ☐ Yes  ☐ No  ☐ Not sure |  |
| 3.5 Note: | Open text field |  |
| 3.5.1 If yes, are the emergency evacuation/exit wheelchair accessible? | ☐ Yes  ☐ No  ☐ Not sure |  |
| 3.5.1 Note: | Open text field |  |
| 3.5.2 If yes, are the emergency evacuation/exit routes signed with pictures and Braille? | ☐ Yes  ☐ No  ☐ Not sure |  |
| 3.5.2 Note: | Open text field |  |
| 3.5.3 If yes, are the emergency evacuation/exit routes with warning lights for the deaf? | ☐ Yes  ☐ No  ☐ Not sure |  |
| 3.5.2 Note: | Open text field |  |
| 3.6 Are the corridors and room floors made of non-slip materials? | ☐ Yes  ☐ No  ☐ Not sure |  |
| 3.6 Note: | Open text field |  |
| 3.7 Are all service rooms on the same level? | ☐ Yes  ☐ No  ☐ Not sure |  |
| 3.7 Note: | Open text field |  |
| 3.7.1 If no, are there functional lifts or ramps if some service rooms are on on upper floors? | ☐ Yes  ☐ No  ☐ Not sure |  |
| 3.7.1 Note: | Open text field |  |
| 3.8 Is the examination table height adjustable to suit people with different requirements (e.g. wheelchair users, people of short stature) | ☐ Yes  ☐ No  ☐ Not sure |  |
| 3.8 Note: | Open text field |  |
| 3.9 Is there any accessible bed at the facility? | ☐ Yes  ☐ No  ☐ Not sure |  |
| 3.9 Note: | Open text field |  |
| 3.10 Are caretakers/support persons welcomed and allowed to support people with disabilities in the process of accessing care? | ☐ Yes  ☐ No  ☐ Not sure |  |
| 3.10 Note: | Open text field |  |
| **Universal design and accessibility - Toilets** | |  |
| 4.0 Is there a designated disability accessible toilet/latrine? | ☐ Yes  ☐ No  ☐ Not sure |  |
| 4.0 Note: | Open text field |  |
| 4.0.1 If yes, is it clearly labelled as disability accessible? | ☐ Yes  ☐ No  ☐ Not sure |  |
| 4.0.1 Note: | Open text field |  |
| 4.0.2 If yes, what is the usable width of the toilet door for the disability accessible toilet/latrine? | *Measurement (cm)* |  |
| 4.0.3 If yes, are there wash points that a wheelchair user can reach? | ☐ Yes  ☐ No  ☐ Not sure |  |
| 4.0.3 Note: | Open text field |  |
| 4.0.4 If yes, are there hand rails? | ☐ Yes  ☐ No  ☐ Not sure |  |
| 4.0.4 Note: | Open text field |  |
| Note | This section is about Reasonable Accommodation. By Reasonable Accommodation, we mean whether the facility provides modifications and adjustments to support people with disabilities specifically |  |
| **Reasonable accommodation of people with disabilities - Information and communication** | |  |
| 5.0 Does the facility provide any alternative communication techniques for people who require it for example sign language or braille? | ☐ Yes  ☐ No  ☐ Not sure |  |
| 5.0 Note: | Open text field |  |
| 5.0.1 If yes, please specify: | ☐ Sign Language Interpretation  ☐ Writing down instructions  ☐ Other |  |
| 5.0.1a If other communication technique, specify: | Open text field |  |
| 5.1 Does the facility provide any written health information for patients e.g. a vaccination leaflet? | ☐ Yes  ☐ No  ☐ Not sure |  |
| 5.1 Note: | Open text field |  |
| 5.1.1 If yes, does the facility provide any alternative formats for health information for people with disabilities who require it? | ☐ Yes  ☐ No  ☐ Not sure |  |
| 5.1.1 Note: | Open text field |  |
| 5.1.2 If yes, please specify: | ☐ Braille  ☐ Braille readable format  ☐ Text speech compatible format  ☐ Audio recorded format  ☐ large print  ☐ simplified  ☐ pictorial  ☐ Other |  |
| 5.1.2.1 If any other alternative formats, specify: | Open text field |  |
| 5.2 Does the facility provide any written informed consent sheets for patients? | ☐ Yes  ☐ No  ☐ Not sure |  |
| 5.2 Note: | Open text field |  |
| 5.2.1 If yes, does the facility provide any alternative formats for informed consent for people with disabilities who require it? | ☐ Yes  ☐ No  ☐ Not sure |  |
| 5.2.1 Note: | Open text field |  |
| 5.2.2 If yes, please specify: | ☐ Braille  ☐ Braille readable format  ☐ Text speech compatible format  ☐ Audio recorded format  ☐ large print  ☐ simplified  ☐ pictorial  ☐ Other |  |
| 5.2.2.1 If any other alternative consent formats, specify: | Open text field |  |
| 5.3 Does the facility provide information on medicines and where to access them for patients? | ☐ Yes  ☐ No  ☐ Not sure |  |
| 5.3 Note: | Open text field |  |
| 5.3.1 If yes, does the facility provide information about more convenient dispensary options that are accessible and available to people with disabilities? | ☐ Yes  ☐ No  ☐ Not sure |  |
| 5.3.1 Note: | Open text field |  |
| **Reasonable accommodation of people with disabilities - Assistance and support** | |  |
| 6.0 Is there assistance available to fill in forms and documents (paper or electronic) if needed? | ☐ Yes  ☐ No  ☐ Not sure |  |
| 6.0 Note: | Open text field |  |
| 6.1 Are there functional assistive devices available for temporary use (wheelchairs, crutches, walkers, buggies) if required? | ☐ Yes  ☐ No  ☐ Not sure |  |
| 6.1 Note: | Open text field |  |
| 6.2 Are guiding services available if required e.g. is there someone who can take the person with a disability around the facility? | ☐ Yes  ☐ No  ☐ Not sure |  |
| 6.2 Note: | Open text field |  |
| 6.3 Is there a way for people to make complaints or suggestions e.g. suggestion boxes | ☐ Yes  ☐ No  ☐ Not sure |  |
| 6.3 Note | Open text field |  |
| 6.3.1 If yes to 6.3, is it available in any alternative format for people with disabilities if required? | ☐ Yes  ☐ No  ☐ Not sure |  |
| 6.3.1 Note: | Open text field |  |
| 6.4 Does the facility have routine patient satisfaction surveys? | ☐ Yes  ☐ No  ☐ Not sure |  |
| 6.4 Note: | Open text field |  |
| 6.4.1 If yes, are they available in accessible format for people with disabilities if required? | ☐ Yes  ☐ No  ☐ Not sure |  |
| 6.4.1 Note: | Open text field |  |
| 6.5 There is clear signage indicating that people with disabilities will be moved to the front of the queue if required | ☐ Yes  ☐ No  ☐ Not sure |  |
| 6.5 Note: | Open text field |  |
| **Capacity of facility staff to identify and support people with disabilities** | |  |
| 7.0 Is there a disability focal person at the facility? | ☐ Yes  ☐ No  ☐ Not sure |  |
| 7.0 Note: | Open text field |  |
| 7.0.1 If yes, has that individual been provided training in supporting patients with disabilities? | ☐ Yes  ☐ No  ☐ Not sure |  |
| 7.0.1 Note: | Open text field |  |
| 7.1 Have any staff members been trained on disability accessibility issues? | ☐ Yes  ☐ No  ☐ Not sure |  |
| 7.1 Note: | Open text field |  |
| 7.2 Have all staff in all cadres been trained on disability etiquette or sensitisation (including accessibility?) | ☐ Yes  ☐ No  ☐ Not sure |  |
| 7.2 Note: | Open text field |  |
| 7.3 Has training been conducted on supporting emergency evacuation for people of disabilities (at least one staff member on duty) | ☐ Yes  ☐ No  ☐ Not sure |  |
| 7.3 Note: | Open text field |  |
| 7.4 Has training been conducted on basic sign language interpretation and Braille signage (at least one staff member on duty) | ☐ Yes  ☐ No  ☐ Not sure |  |
| 7.4 Note: | Open text field |  |
| 7.5 Has there been any training conducted on Sexual Reproductive Health and Rights (SRHR) and gender-based violence (GBV)? | ☐ Yes  ☐ No  ☐ Not sure |  |
| 7.5 Note: | Open text field |  |
| 7.5.1 If yes to 7.5, did it include people with disabilities? | ☐ Yes  ☐ No  ☐ Not sure |  |
| 7.5.1 Note | Open text field |  |
| 7.6 Has training been conducted on disability-related violence (e.g. name- calling, taking away of assistive devices) (at least one staff member on duty) | ☐ Yes  ☐ No  ☐ Not sure |  |
| 7.6 Note: | Open text field |  |
| 7.7 Has training been conducted for healthcare workers on how to screen for and identify disability (E.g., mental, intellectual, physical, hearing and visual) | ☐ Yes  ☐ No  ☐ Not sure |  |
| 7.7 Note: | Open text field |  |
| 7.8 Has training been conducted for healthcare workers on how to refer for rehabilitation/specialized hospital services e.g. physiotherapy or occupational therapy? | ☐ Yes  ☐ No  ☐ Not sure |  |
| 7.8 Note: | Open text field |  |
| 7.9 Has training been conducted for healthcare workers on awareness and use of assistive devices e.g. wheelchairs, white cane? | ☐ Yes  ☐ No  ☐ Not sure |  |
| 7.9 Note: | Open text field |  |
| 7.10 Do service protocols or guidelines incorporated specific guidance on addressing the needs of people with disability? | ☐ Yes  ☐ No  ☐ Not sure |  |
| 7.10 Note: | Open text field |  |
| 7.11 Are there any health promotional and educational materials? | ☐ Yes  ☐ No  ☐ Not sure |  |
| 7.11 Note: | Open text field |  |
| 7.11.1 If yes to 7.10, are these materials in accessible format for people with disabilities if required? | ☐ Yes  ☐ No  ☐ Not sure |  |
| 7.11.1 Note: | Open text field |  |
| **Linkage to disability and rehabilitation services** | |  |
| 8.0 Are there registers that capture disability status and needed accommodations? | ☐ Yes  ☐ No  ☐ Not sure |  |
| 8.0 Note: | Open text field |  |
| 8.1 Are there screening tools available and used to identify impairments for children and adults (e.g. physical, mental, visual, hearing, intellectual)? | ☐ Yes  ☐ No  ☐ Not sure |  |
| 8.1 Note: | Open text field |  |
| 8.2 Do routine data summaries include people with disabilities served by the facility? | ☐ Yes  ☐ No  ☐ Not sure |  |
| 8.2 Note: | Open text field |  |
| 8.3 Is there a referral and follow up monitoring tool? | ☐ Yes  ☐ No  ☐ Not sure |  |
| 8.3 Note: | Open text field |  |
| 8.4 Is there an annually updated referral process to disability services providing assistive devices? | ☐ Yes  ☐ No  ☐ Not sure |  |
| 8.4 Note: | Open text field |  |
| 8.5 Is there an annually updated referral and follow-up system for repairing assistive devices? | ☐ Yes  ☐ No  ☐ Not sure |  |
| 8.5 Note: | Open text field |  |
| 8.6 Is there an annually updated referral process to rehabilitation/specialized medical services (E.g. Occupational Therapy, Speech Therapy, Audiology, Physiotherapy, Podiatry and Psychology)? | ☐ Yes  ☐ No  ☐ Not sure |  |
| 8.6 Note: | Open text field |  |
| 8.7 Is there an annually updated referral process to mental healthcare providers/services accessible to people with disabilities? | ☐ Yes  ☐ No  ☐ Not sure |  |
| 8.7 Note: | Open text field |  |
| 8.8 Is there an annually updated directory of organizations for people with disabilities available at the facility for ease of access to additional support services? | ☐ Yes  ☐ No  ☐ Not sure |  |
| 8.8 Note: | Open text field |  |
| 8.9 Are there linkages and active collaborations with organisations for people with disabilities? | ☐ Yes  ☐ No  ☐ Not sure |  |
| 8.9 Note: | Open text field |  |
| 8.10 Are there linkages with a rehabilitation outreach programme that visits the facility at least every 6 months? | ☐ Yes  ☐ No  ☐ Not sure |  |
| 8.10 Note: | Open text field |  |
| 8.11 Are there linkages and referral to Home-Based or Community- Based Rehabilitation programmes in the area? | ☐ Yes  ☐ No  ☐ Not sure |  |
| 8.11 Note: | Open text field |  |
| 9.9 Filled DAC health facility feedback and recommendation form | Image upload |  |
| **Facility GPS Location and form completion** | |  |
| 9.0 Location of health facility (Latitude): | GPS Input |  |
| 9.1 Location of health facility (Longitude): | GPS Input |  |
| 9.2 Location of nearest boda stage (Latitude): | GPS Input |  |
| 9.3 Location of nearest boda stage (Longitude): | GPS Input |  |
| 9.4 Location of nearest taxi route (Latitude): | GPS Input |  |
| 9.5 Location of nearest taxi route (Longitude): | GPS Input |  |
| 9.6 Audit end time: | Time input |  |
| 9.7 Audit duration: | Calculation |  |
| 9.8 General observations (comments): | Open text field |  |

## Appendix 3: Disability Access checklist facility feedback template

| **Key discoveries/comments** | **Key Suggestions/recommendations** |
| --- | --- |
| A). **Universal Design and accessibility-Entrance** | |
| 1)……………………………………………………  ……………………………………………………  ………………………………………………………  2)……………………………………………………  ……………………………………………………  ………………………………………………………  3)……………………………………………………  ……………………………………………………… | 1)…………………………………………………………  ……………………………………………………………  ……………………………………………………………  2)…………………………………………………………  ……………………………………………………………  …………………………………………………………….  3)…………………………………………………………  …………………………………………………………….. |
| **B) Universal design and accessibility-Reception, corridors and waiting rooms** | |
| 1)……………………………………………………  ……………………………………………………  ………………………………………………………  2)……………………………………………………  ……………………………………………………  ………………………………………………………  3)……………………………………………………  ……………………………………………………… | 1)…………………………………………………………  ……………………………………………………………  ……………………………………………………………  2)…………………………………………………………  ……………………………………………………………  …………………………………………………………….  3)…………………………………………………………  …………………………………………………………….. |
| **C) Universal design and accessibility- Latrines/toilets** | |
| 1)……………………………………………………  ……………………………………………………  ………………………………………………………  2)……………………………………………………  ……………………………………………………  ………………………………………………………  3)……………………………………………………  ……………………………………………………… | 1)…………………………………………………………  ……………………………………………………………  ……………………………………………………………  2)…………………………………………………………  ……………………………………………………………  …………………………………………………………….  3)…………………………………………………………  …………………………………………………………….. |
| **D). Reasonable Accommodation of people with disabilities-information and communication** | |
| 1)……………………………………………………  ……………………………………………………  ………………………………………………………  2)……………………………………………………  ……………………………………………………  ………………………………………………………  3)……………………………………………………  ……………………………………………………… | 1)…………………………………………………………  ……………………………………………………………  ……………………………………………………………  2)…………………………………………………………  ……………………………………………………………  …………………………………………………………….  3)…………………………………………………………  …………………………………………………………….. |
| **E) Reasonable Accommodation of people with disabilities-Assistance and support** | |
| 1)……………………………………………………  ……………………………………………………  ………………………………………………………  2)……………………………………………………  ……………………………………………………  ………………………………………………………  3)……………………………………………………  ……………………………………………………… | 1)…………………………………………………………  ……………………………………………………………  ……………………………………………………………  2)…………………………………………………………  ……………………………………………………………  …………………………………………………………….  3)…………………………………………………………  …………………………………………………………….. |
| **F) Capacity of Staff to identify and support People with disabilities** | |
| 1)……………………………………………………  ……………………………………………………  ………………………………………………………  2)……………………………………………………  ……………………………………………………  ………………………………………………………  3)……………………………………………………  ……………………………………………………… | 1)…………………………………………………………  ……………………………………………………………  ……………………………………………………………  2)…………………………………………………………  ……………………………………………………………  …………………………………………………………….  3)…………………………………………………………  …………………………………………………………….. |
| **G) Linkage to disability and Rehabilitation services** | |
| 1)……………………………………………………  ……………………………………………………  ………………………………………………………  2)……………………………………………………  ……………………………………………………  ………………………………………………………  3)……………………………………………………  ……………………………………………………… | 1)…………………………………………………………  ……………………………………………………………  ……………………………………………………………  2)…………………………………………………………  ……………………………………………………………  …………………………………………………………….  3)…………………………………………………………  …………………………………………………………….. |
| **DAC and feedback conducted by**  Name……………………………………………….  Date………………………………………………...  Signature…………………………………………… | |

**References**

1. Turyamureba M, Yawe B, Oryema JB. Health Care Delivery System in Uganda: a review. Tanzania Journal of Health Research. 2023 Mar 26;24(2):57–64.

2. Mays DC, O’Neil EJ, Mworozi EA, Lough BJ, Tabb ZJ, Whitlock AE, et al. Supporting and retaining Village Health Teams: an assessment of a community health worker program in two Ugandan districts. International Journal for Equity in Health. 2017 Jul 20;16(1):129.

3. Source [Internet]. 2014 [cited 2024 Feb 20]. Accessibility standards : a practical guide to create a barrier-free physical environment in Uganda. Available from: https://asksource.info/resources/accessibility-standards-a-practical-guide-create-a-barrier-free-physical-environment

4. Azuba Ntege MHon. The Building Control (Accessibility Standards for Persons with Disabilities) Code, 2019 [Internet]. Report No.: 2019 No. 52. Available from: https://www.kcca.go.ug/media/docs/The%20Building%20Control%20(Accessibilty%20Standards%20For%20Persons%20with%20Disabilities)%20Code,%202019.pdf
